# High speed sCMOS‐based oblique plane microscopy applied to the study of calcium dynamics in cardiac myocytes

**DOI:** 10.1002/jbio.201500193

**Published:** 2015-10-21

**Authors:** Markus B. Sikkel, Sunil Kumar, Vincent Maioli, Christina Rowlands, Fabiana Gordon, Sian E. Harding, Alexander R. Lyon, Kenneth T. MacLeod, Chris Dunsby

**Affiliations:** ^1^ Myocardial Function Section, National Heart and Lung InstituteImperial CollegeLondonUnited Kingdom; ^2^ Photonics Group, Department of PhysicsImperial College LondonLondonUnited Kingdom; ^3^ Statistics Advisory ServiceImperial College LondonLondonUnited Kingdom; ^4^ NIHR Cardiovascular Biomedical Research UnitRoyal Brompton HospitalLondonUnited Kingdom; ^5^ Centre for Pathology, Department of MedicineImperial College LondonUnited Kingdom

**Keywords:** high‐speed imaging, light‐sheet microscopy, calcium dynamics, 3D imaging, Oblique Plane Microscopy

## Abstract

Oblique plane microscopy (OPM) is a form of light sheet microscopy that uses a single high numerical aperture microscope objective for both fluorescence excitation and collection. In this paper, measurements of the relative collection efficiency of OPM are presented. An OPM system incorporating two sCMOS cameras is then introduced that enables single isolated cardiac myocytes to be studied continuously for 22 seconds in two dimensions at 667 frames per second with 960 × 200 pixels and for 30 seconds with 960 × 200 × 20 voxels at 25 volumes per second. In both cases OPM is able to record in two spectral channels, enabling intracellular calcium to be studied via the probe Fluo‐4 AM simultaneously with the sarcolemma and transverse tubule network via the membrane dye Cellmask Orange. The OPM system was then applied to determine the spatial origin of spontaneous calcium waves for the first time and to measure the cell transverse tubule structure at their point of origin. Further results are presented to demonstrate that the OPM system can also be used to study calcium spark parameters depending on their relationship to the transverse tubule structure.

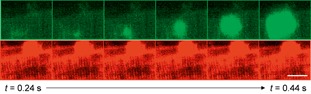

## 1 Introduction

Calcium (Ca^2+^) dynamics within cardiac myocytes include Ca^2+^ sparks, spontaneous Ca^2+^ waves and stimulated global Ca^2+^ transients. Ca^2+^ sparks, which are the elemental building blocks of larger events such as transients and waves, are both spatially and temporally confined 1, 2, 3. Ca^2+^ waves propagate through the cell and result in depolarisations (so‐called “delayed after‐depolarisations”) that may, in turn, trigger arrhythmias within the heart. Therefore, studying the elementary release events and the formation of waves is essential to understanding the aetiology of malignant cardiac arrhythmias such as ventricular tachycardia and ventricular fibrillation, which are responsible for approximately 50% of deaths in patients with heart failure (HF) 4, 5. Cardiac myocytes contain invaginations of the cell membrane known as transverse tubules (*t*‐tubules) that are essential for the normal process of Ca^2+^‐induced Ca^2+^ release. We and others have previously reported that the organization of *t*‐tubule networks becomes significantly deranged in HF in both failing human hearts and animal models, in part due to a reduction in *t*‐tubule density (detubulation) 6, 7, 8, 9, 10. Whether these structural changes and enhanced arrhythmogenesis via an increase in Ca^2+^ waves are linked is difficult to elucidate using current microscopy techniques.

Confocal microscopy provides optically sectioned imaging of the specimen and is usually required in order to reliably detect Ca^2+^ sparks 1 in isolated single cardiac myocytes. However, since a high temporal resolution is also required this imaging is usually performed in line‐scanning mode whereby a single line of the cell is raster scanned to build up a 1‐dimensional (1‐D) image over time (*x*–*t*). This typically allows the line to be scanned every 1–2 ms. Although this technique is useful, Ca^2+^ waves are relatively rare events and therefore in order to ascertain their origin, high speed 3‐D fluorescence microscopy is required. In addition the limited spatial information results in skewed measurements of spark morphology 11.

Currently, the fastest optically sectioning 2‐D microscopy techniques for sub‐cellular imaging include multi‐beam confocal microscopes 12 and slit‐scanning confocal microscopes 13, 14. Slit‐scanning confocal microscopy has been used to investigate spark properties in time‐lapse 2‐D imaging (*x*‐*y*‐*t*) at up to 670 fps with 512 × 30 pixels 13. Recently, this approach has been combined with rapid axial scanning to achieve time‐lapse imaging of trios of image planes, where three 512 × 31 pixel images separated by 1 micron in the vertical direction were acquired within 5.6 ms 15.

Another approach to high speed 2‐D and 3‐D sub‐cellular imaging of isolated single cells is oblique plane microscopy (OPM). OPM is a light sheet microscopy technique that uses the same high NA microscope objective to provide both the fluorescence illumination and detection 16, 17. OPM can therefore be implemented on a standard inverted fluorescence microscope and is compatible with a range of conventional sample mounting techniques including coverslip and microscope slide, multi‐well plates or superfusion system. A comparison of OPM to other light sheet microscopy techniques can be found in the introduction sections of references 16, 17 and the following review articles 18, 19.

In order to usefully apply OPM to the study of Ca^2+^ dynamics in live cells, a system employing multiple excitation laser lines and two high‐speed scientific complementary metal–oxide–semiconductor (sCMOS) cameras was developed and applied to imaging cardiac myocytes in two spectral channels simultaneously with a pixel/voxel rate of ˜10^8^ s^–1^. This system was then used to achieve video‐rate time‐lapse 3‐D imaging of spontaneous Ca^2+^ waves and enabled their spatial origins in 3‐D to be determined and correlated with local *t*‐tubule structure. In this initial study on a small number of cells, the results show that in heart failure the majority of spontaneous Ca^2+^ waves originate from regions of the cell where the periodic modulation in fluorescence intensity due to the *t*‐tubule structure, which we refer to as *t*‐tubule organization, is high. High‐speed 2‐D OPM was then used to study regional differences in spark characteristics within cells and the results were analysed using hierarchical statistics. This study therefore exemplifies the use of high‐speed 2‐D and 3‐D OPM to acquire structural and functional information from single cardiac myocytes.

## 2 Methods

### 2.1 Animal model

The rat post myocardial infarction HF model was generated as described previously 7. All studies were carried out with the approval of the local Imperial College ethical review board and the Home Office, UK, under project licenses 70/6568 and 70/7399. Animal surgical procedures and perioperative management were carried out in accordance with the United Kingdom Home Office Guide on the Operation of the Animals (Scientific Procedures) Act 1986, which conforms to the Guide for the Care and Use of Laboratory Animals published by the U.S. National Institutes of Health under assurance number A5634‐01. Animals were anaesthetised using 5% isoflurane, which was reduced to 1.5% once they were intubated and ventilated. Pre‐operative medication that was administered consisted of: 0.015–0.03 mg buprenorphine (0.05–0.1 mg/kg), 1.25–1.5 mg enrofloxacin (5 mg/kg), and 2.5 ml 0.9% saline subcutaneously. Post‐operative pain management consisted of repeated buprenorphine administration as required. M‐mode echocardiography was performed for phenotyping using a Vevo 770 system to assess left ventricular dimensions and blood flow via pulsed wave Doppler measurements of the pulmonary artery.

To validate this model, phenotypic parameters were compared for 6 AMC and 8 HF hearts. A statistically significant increase in heart weight : body weight ratio (*p* = 0.0011) and reduced ejection fraction (*p* < 0.0001) were present in HF animals. Left ventricular internal diameter in diastole was significantly increased in HF (*p* = 0.0001) and maximal velocity of blood in the pulmonary artery was significantly reduced (*p* = 0.003). Together these measurements revealed the presence of cardiac dilatation and global contractile dysfunction which are expected in HF. Spontaneous Ca^2+^ wave frequency was assessed in isolated cardiomyocytes in cells loaded with Fura‐2AM as described previously 20 in *n* = 76 AMC cells from 10 animals and 79 HF cells from 10 animals. A statistically significant increase in spontaneous Ca^2+^ wave frequency was seen in cells from HF rats compared with cells from (AMC 0.029 ± 0.003; HF 0.016 ± 0.003 waves · s^–1^; *p* = 0.023). This difference in wave frequency was not due to prevailing diastolic cytoplasmic [Ca^2+^] as measured by Fura‐2 ratio, which was similar in AMC and HF (*p* = 0.98).

### 2.2 Sample preparation for OPM imaging

Cells were first incubated with 15 µM Fluo‐4 AM and 0.16% pluronic acid for 25 mins at room temperature. Cellmask Orange at a concentration of 5 µg · ml^–1^ was then added to this solution for 5 mins with the temperature increased to 37 °C. The suspension of myocytes was mixed using a rotary mixer and protected from light during the incubation period. Cells were then washed twice and resuspended in a low Ca^2+^ form of normal tyrode (NT) as above.

Cells were attached to coverslips using mouse laminin (Sigma‐Aldrich) and superfused with Normal Tyrode (NT) containing (in mM): NaCl (140), KCl (6), glucose (10), HEPES (10), MgCl_2_ (1), CaCl_2_ (2), pH adjusted to 7.4 with NaOH. Cells were field stimulated via platinum electrodes at 1.5× threshold voltage. Cells were field stimulated prior to imaging for at least 2 mins at 1 Hz.

### 2.3 OPM system

**Figure 1:**
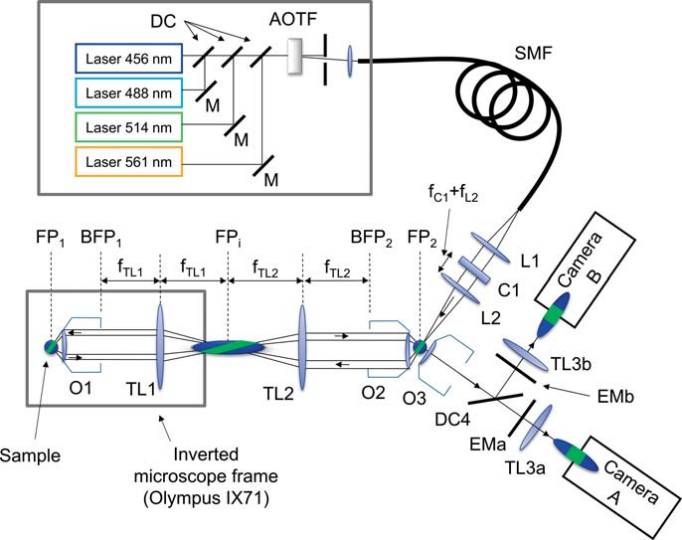
Experimental configuration for the OPM system illustrated with a fluorescent sphere (blue) placed in the sample plane. The region in the sphere where fluorescence is excited is shown in green. The image of the sphere is illustrated at the image planes in the optical system. See text for further detail. M – mirror; DC – dichroic beamsplitter; AOTF – acousto‐optic tunable filter; SMF – single mode optical fibre; L – spherical lens; C – cylindrical lens; FP – front focal plane; BFP – back focal plane; O – microscope objective; TL – microscope tube lens; EM – emission filter.

The OPM system was based around an inverted microscope (Olympus IX71) that allowed imaging of isolated cardiomyocytes in a low volume gravity driven superfusion chamber (Warner RC‐24N) providing rapid solution flow at 37 °C 20. OPM uses a single high numerical aperture microscope objective (O1 in Figure 1) to both deliver a tilted sheet of excitation light to the sample and collect the resulting fluorescence emission. It consists of three separate microscopes placed in series. The first microscope (O1 and TL1 [tube lens1] in Figure 1) is the conventional inverted microscope frame and the second (O2 and TL2) is used to produce an intermediate image of the specimen (FP_2_ in Figure 1) where the lateral and axial magnifications are equal. A third microscope (O3 and TL3) images a tilted plane within the intermediate image onto sCMOS cameras. The plane of observation of the third microscope is aligned to overlap the excitation sheet within the sample. The angle between the optical axes of O2 and O3 (the OPM angle) was set at 35°.

The system used in the current study includes continuous wave laser sources operating at 457 nm (Twist‐25, Cobolt AB), 488 nm (Sapphire 488 LP, Coherent), 514 nm (Fandango‐25, Cobolt AB) and 561 nm (Jive‐25, Cobolt AB). These sources were combined using dichroic beamsplitters, and their amplitude was controlled using an acousto‐optic tunable filter (AOTFnC‐400.650, AA Optoelectronics). The light was then coupled into a single mode optical fibre. The light emitted from the fibre was collimated by lens L1 in Figure 1 (10× microscope objective, Olympus), focused in the vertical direction by cylindrical lens C1 (*f* = 50 mm) onto the back focal plane of lens L2 (achromatic doublet, *f* = 25 mm), which produced a light sheet at O2 that was tilted at an angle of 35° with respect to the front focal plane of O2 (FP2). An image relay consisting of two microscopes placed back to back formed by microscope objective O2 (50×/0.95 NA, Olympus), tube lenses TL2 (*f* = 162 mm consisting of an *f* = 150 mm achromatic doublet and an *f* = –500 mm aplanatic meniscus separated by 97 mm) and TL1 (*f* = 180 mm, Olympus) and microscope objective O1 (60×/1.2 NA water immersion, Olympus) was used to image the light sheet into the sample. The resulting fluorescence from the sample was then relayed back to FP2 by the same optics. Finally, a plane (coplanar with the illumination light sheet) was imaged by the microscope formed by O3 (40×/0.6 NA, Nikon) and tube lenses TL3a & TL3b (achromatic doublets, *f* = 100 mm) onto two scientific CMOS cameras (sCMOS, PCO.edge, PCO GmbH). The effective pixel size at the sample was 0.24 µm. A dichroic beamsplitter DC4 (T585LP, Chroma) was used to split the fluorescence into two separate spectral detection channels that were further defined by emission filters EMa & EMb (ET630/75M & ET525/50 respectively). The effective axial position of the light sheet and tilted observation plane in the sample was determined by the axial position of O2, which was controlled using a piezo‐electric objective actuator (Physik Instrumente, Germany, part number P‐721.CLQ with controller E‐501.00 using amplifier E‐505.00 and sensor control module E‐509.C1A). Custom‐written software running in LabVIEW was used to provide synchronized control of the AOTF, piezo‐electric objective actuator and camera acquisition.

The critical alignment steps are that the pupil of O1 should be accurately imaged onto the pupil of O2 and that the total magnification provided by microscope 1 (O1 and TL1) and microscope 2 (O2 and TL2) should be equal in the lateral and axial directions, see 21. The lateral magnification of microscope 2 can be adjusted by controlling the separation of the achromatic doublet and meniscus lenses forming TL2 17. In addition, as the fluorescence image is brought to an intermediate focus between O2 and O3, it is important that the whole setup is mounted on a vibration isolated optical table, as any lateral vibration of the position of O2 or O3 causes the final image to vibrate also. As with any optical imaging system, it is corrected to work for a specific sample refractive index and any variation in the sample refractive index from the design value will result in aberrations.

Fluo‐4 and CMO were excited using wavelengths of 488 nm and 561 nm respectively. The corresponding excitation powers at the back aperture of O1 were ˜280 µW for each wavelength. This power was distributed over an illumination sheet covering the full width of the field of view of the system (limited to 400 µm by O2), which is larger than the field imaged by the sCMOS camera of 234 × 49 µm.

Each 2‐D acquisition consisted of 15,000 frames of 960 × 200 pixels on each sCMOS camera. The camera integration time was set at 1.46 ms and the time between frames was 1.5 ms, resulting in an image acquisition rate of 667 frames per second for a total duration of 22.5 s. The two image stacks representing the different channels were acquired simultaneously and then co‐registered using correction parameters determined from an image of a USAF 1951 test chart (Edmund Optics, Barrington, USA).

3‐D data acquisition consisted of 750 volumes of 960 × 200 × 20 voxels on each sCMOS camera. The camera integration and time between frames was the same as for 2‐D imaging. During this acquisition, the position of the piezo‐electric actuator was used to axially translate O2. The actuator was driven with an asymmetric saw‐tooth motion profile with a 30 ms time period where the commanded axial position increased and then a 10 ms time period to allow fly‐back of the piezo‐electric actuator to its initial position ready for the next volume. The amplitude of the commanded motion range depended on the size of the cell being imaged and the maximum axial scan range of O2 was 77 µm. The actual position of O2 was recorded by a capacitive position sensor, see example in supporting information Figure S1, and this information was used for volume transformation during image processing. Overall, this resulted in an acquisition rate of 25 volumes per second. Co‐registration of the image data from the two detection channels was performed in the same way as for 2‐D data.

In order to demonstrate the temporal stability of the OPM system during time‐lapse 3‐D imaging, we imaged a volume of fluorescent beads (TetraSpeck™ Microspheres, 0.2 µm, fluorescent blue/green/orange/dark red, ref. T‐7280) mounted in agarose. The acquisition was performed at 25 volumes per second for a duration of 30 s over an axial scan range of O2 of 77 µm with the same parameters as described above. We then took three orthogonal 2‐D slices through this 4‐D data set passing through the central position of a single isolated fluorescent bead, see supporting information Figure S2. By taking the centre‐of‐mass of these plots as a function of time we found that the standard deviation of the measured bead centre of mass was 15 nm and 13 nm in the plane of the light sheet and 23 nm perpendicular to the plane of the light sheet, which are all well below the optical resolution of the system. The plots in supporting information Figure S2 show no evidence of unwanted higher frequency mechanical resonances of the microscope objective or piezo actuator.

### 2.4 Time‐lapse 3‐D data analysis

Co‐registered 3D datasets were transformed using custom written software in MATLAB utilizing the *tformarray* function to resample the data using linear interpolation from a stack of points lying on oblique planes to a conventional Cartesian *x*–*y*–*z*‐coordinate set where *z* is along the optical axis. Calls to this function were implemented in parallel to reduce the time required for data processing.

The time‐lapse volumetric Fluo‐4 data was then viewed manually and the coordinates of the origin of each spontaneous Ca^2+^ wave was recorded.

A commonly used method of detecting *t*‐tubule structure is binarisation of *t*‐tubule images using the method proposed by Otsu 22, which works on maximising the separation of the pixel values in the two segmented regions. This approach did not work well for our data, which we attribute to the bright sarcolemmal staining compared to *t*‐tubules. We therefore pursued a Fourier domain filtering technique that takes advantage of the periodic nature of transverse tubules. Our approach was applied uniformly to all images with no user input other than to identify the location of the peak in Fourier space due to transverse tubules.

**Figure 2:**
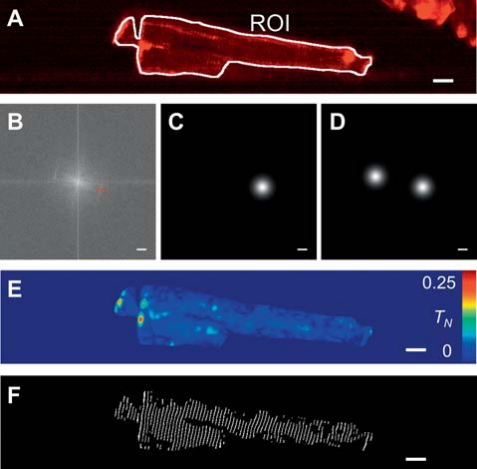
Identification of *t*‐tubule locations within cardiomyocytes. (**A**) CMO image of a single isolated MI cardiac myocyte. Red shows the fluorescence intensity of CMO and white shows the sarcolemmal region of interest (ROI). (**B**) Fourier transform of image shown in A. Red cross indicates position of manually located high spatial frequency corresponding to *t*‐tubule structure. (**C**) and (**D**) one‐ and two‐sided Gaussian spatial‐frequency filters respectively. (**E**) calculated map of normalised *t*‐tubule modulation *M_N_*. (**F**) Binary map of calculated *t*‐tubule locations. Scale bars in (**A**), (**E**) and (**F**) represent 10 µm. Scale bars in (**B**), (**C**) & (**D**) represent a spatial frequency of 0.25 µm^–1^.

For each wave origin, the sarcolemma visualised in the CMO channel was manually selected, see Figure 2A, for each *z*‐plane of the volume, thus producing a region of interest (ROI) defining the extent of the interior of the cell in 3‐D. The *z*‐plane corresponding to the centre of the cell was then found and the region of the image inside the ROI was Fourier transformed and the centre of the high spatial frequency peak arising from the periodic *t*‐tubule structure was selected manually (*u_t_*, *v_t_*) (Figure 2B). A one‐sided high pass filter centred on this peak (*u_t_*, *v_t_*) using a Gaussian window with a full width at half maximum of 0.35 µm^–1^ (Figure 2C) was then applied to the Fourier transform of the image region inside the ROI for each *z*‐plane in the stack. The one‐sided filtered data was then inverse Fourier transformed and the absolute value of the resulting complex image was taken, yielding the *t*‐tubule modulation map *T* (Figure 2E). A two‐sided high‐pass spatial frequency filter (Figure 2D) of the CMO image with Gaussian windows centred at (*u_t_*, *v_t_*) and (–*u_t_*, –*v_t_*) in spatial frequency space was also generated to use in later steps (see below).

So that nuclei were not incorrectly defined as a detubulated cytosolic region, each cell was manually assessed for the presence of a nucleus within the section imaged. The nucleus was identified by its characteristic ellipsoid shape and the presence of a prolonged Ca^2+^ transient during evoked contractions as well as their location corresponding spatially to the nucleus identified on the transmission image of each cell. These regions were excluded from further analysis.

### 2.5 Time‐lapse 2‐D data analysis

Sparks were automatically assessed using custom written software in MATLAB to assess spark morphology in 2‐D, see supporting information S3 for full details. Our spark detection algorithm uses the threshold based algorithm of Cheng et al. 23 and Song et al. 24 but is extended to the analysis of 2‐D time‐lapse (*x*–*y*–*t*) datasets as demonstrated previously 25.

In order to assess the *t*‐tubule organisation at the spatial location of every spark, we averaged the 1000 frames of the CMO channel data and used this image to calculate the *t*‐tubule organisation map *T* as described above. The normalised *t*‐tubule organization *T_N_* was then calculated by dividing *T* by the average fluorescence intensity of the sarcolemma, ⟨*I*_membrane_⟩. ⟨*I*_membrane_⟩ is a measure of how effectively a given cell has been labelled by the CMO and was found by growing the centre line of the sarcolemma (Figure 2A, ROI) by 3 pixels in both directions (resulting in a sarcolemma mask that is 6 pixels/1.4 µm wide) and the mean fluorescence intensity from the resulting region was calculated.

In order to generate a binary mask of *t*‐tubule locations, the normalised *t*‐tubule modulation image *T_N_* (Figure 2E) was thresholded. Regions with *M_N_* > 0.015 were defined to be tubulated and the same threshold was applied to all cells imaged. The *t*‐tubule map was generated by applying the two‐sided high‐pass spatial frequency filter (Figure 2D) to the CMO image and taking the real value of the result. This filtered image was masked by the *t*‐tubule locations and binarised using a threshold of zero. The resulting mask has a mark‐space ratio of 1 : 1 and the *t*‐tubule regions were eroded using MATLAB's imerode function by 0.49 µm (2 pixels). This process results in an image where a binary *t*‐tubule mask is produced for regions where the peak‐to‐trough value of the *t*‐tubules is more than 6% of the mean value of the membrane intensity (Figure 2F). The same data as shown in Figure 2f is shown in supporting information S4 where the image has been adjusted to show that the detected *t*‐tubules lie along the top of the features in the original CMO image.

The calculated centre of mass for each identified spark allowed both the organization of *t*‐tubules in its vicinity (normalized modulation) and its distance to the nearest *t*‐tubule to be determined. Within tubulated regions the centre of mass of each spark was compared to the location of the nearest *t*‐tubule. Epitubular sparks were defined as on the *t*‐tubule (within a margin of error of 1 pixel or approx. 0.25 µm either side), paratubular sparks were those from a region populated with *t*‐tubules but more than 0.25 µm from the nearest *t*‐tubule.

### 2.6 Statistical modelling and significance testing

Statistical analysis was performed using IBM SPSS statistics. Simple parametric statistics (Student's *t*‐tests) were carried out for biometric and echocardiographic data. Spark data was hierarchical in nature (e.g. multiple cells from a single isolation, multiple sparks from within a single cell), thus linear mixed models are the most appropriate means of analysis 26. Such models take into account the relationships between events which occur within a subject. Part of the assessment of hierarchical models includes estimates of covariance parameters which give an assessment of whether this intra‐subject clustering is significant. For spark data that was not significantly clustered within subjects in the hierarchy and where the distribution was not Gaussian (i.e. wave frequency), standard tests such as a Mann‐Whitney U test was used for comparison.

Subcellular spark frequency was normalised to area and time, giving final units of sparks/100 µm^2^/s. The logarithmic transformations of morphological characteristics were used to produce more normally distributed data. These are referred to as LogAmp, LogArea and LogFDHM for the logarithm of spark amplitude, area and full duration at half maximum (FDHM) respectively. Spark frequency and morphological characteristics were the dependent variables in the statistical models. Initial independent variables in each model included regional *t*‐tubule morphology, the presence or absence of HF and the interaction between the two coded as fixed effects. Each model used was assessed for validity by ensuring predicted values closely corresponded to those observed. Residuals were assessed for normality and symmetry. A random intercept was included. Type III tests of fixed effects were used to decide whether variables were significant (*p*‐values pertaining to significance of a parameter refer to this) and non‐significant independent variables were removed from the model stepwise. *P*‐values quoted for significant effects are from the simplified model following removal of non‐significant terms.

## 3 Results

### 3.1 OPM resolution and optical efficiency

The lateral spatial resolution of the optical system was determined by imaging 100 nm fluorescent beads (TetraSpeck™ Microspheres, 0.1 µm, fluorescent blue/green/orange/dark red, ref. T‐7279) embedded in agarose gel approximately 1 mm thick on a coverslip. The 3‐D volume was acquired by scanning the piezo‐electric actuator controlling O2 (see Figure 1) over a range of 100 µm and 100 evenly spaced images were recorded. 17 individual beads were identified in the raw image data and the PSF full‐width at half maximum (FWHM) in the plane of illumination, the FWHM perpendicular to the plane of illumination and the axial position, was determined for each one. The PSF FWHM in the plane of illumination was found to be below 0.5 µm over an axial refocusing range of 65 µm. As the angular acceptance of O1 (NA = 1.2, *n* = 1.33) is 64°, this means that the OPM collection half‐angle can be estimated to be 64 – 35 = 29°, corresponding to a collection NA of 0.64, see Figure 2 of reference 17, and gives a predicted point spread function FWHM of 0.42 µm in reasonable agreement with the measured value.

The illumination sheet FWHM was measured using a thin fluorescent sheet to be 3.8 µm for both 488 nm and 561 nm excitation and the sheet remained below a width of $3.8\times \sqrt{2}$, i.e. the confocal parameter, over a range of 100 µm along the illumination sheet propagation direction. The total radiant exposure to the sample in the direction of the light sheet illumination was therefore estimated to be 4.1 × 10^6^ J · m^–2^ during 2‐D time‐lapse imaging and 2.1 × 10^5^ J · m^–2^ during 3‐D time‐lapse imaging.

The FWHM of the PSF in the direction perpendicular to the illumination plane was measured to be below 2.3 µm over an axial refocusing range of 100 µm. Theoretically, this is determined by the product of the excitation sheet profile and the axial profile of the collection PSF 27. The axial collection PSF FWHM can be estimated using 1.77λ*n*/NA^2^ = 3.0 µm which, if approximated as a Gaussian and combined with the measured illumination sheet thickness, yields a predicted value of 2.4 µm again in reasonable agreement with the measured value.

**Figure 3:**
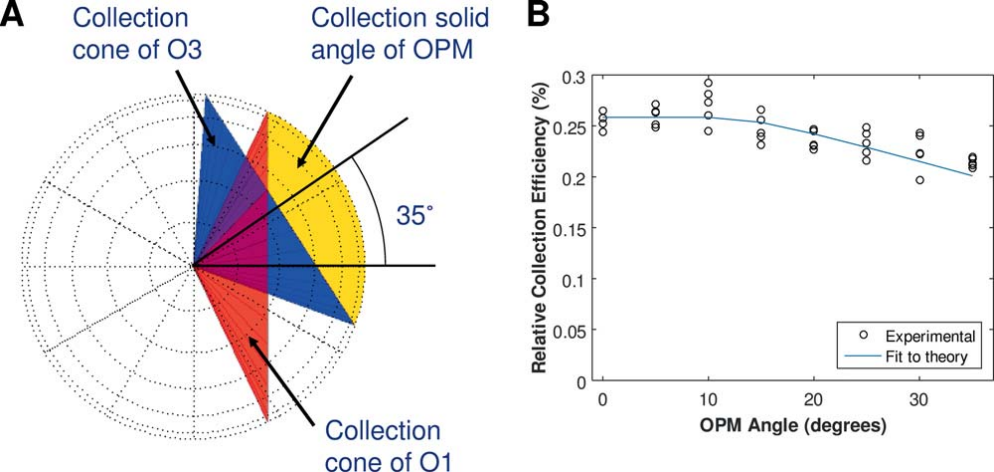
Measurement of the relative collection efficiency of OPM. (**A**) The red cone illustrates the collection solid angle of objective 1 (see Figure 1) on the surface of the unit sphere. The blue cone shows the corresponding collection cone of objective 3 projected onto the same coordinate system. The overlap on the surface of the unit sphere between the two cones is shown in yellow. (**B**) Plot of measured relative OPM collection efficiency as a function of OPM angle together with a fit to the theoretically predicted curve.

The collection efficiency of the OPM system is reduced compared to direct detection of the fluorescence signal in focal plane FP_i_ (see Figure 1) due to two factors. First, only a part of the pupil of O1 is utilised as the pupil aperture of O3 blocks some rays and this geometric transmission factor *t*_geom_ can be calculated by the overlap of two cones on the surface of a unit sphere, see Figure 3A, where the two cones describe the collection cones of O1 and O3. The collection cone of O2 is larger than O1 and so does not limit the geometrical collection efficiency. For an OPM angle of 35°, *t*_geom_ is calculated to be 0.54. The second factor *t*_optics_ is the transmission of the additional optical elements in the detection beam path after FP_i_. This is dominated by the transmission of O2 and O3. The manufacturers' stated transmission for both O2 and O3 is 90% at 520 nm. Including the total transmission of the AR coated tube lenses TL2 and TL3 as 98% (total of 6 surfaces each at ˜99.7%) gives an estimated value of *t*_optics_ as 0.8.

The collection efficiency of the OPM system relative to focal plane FP_i_ (see Figure 1) was measured as a function of the angle between the optical axes of O2 and O3 (the OPM angle) by imaging single 100 nm fluorescent beads placed in the centre of the focal plane of O1. A camera (Andor Luca S) was placed first at FP_i_ and then in the position ’Camera A' with dichroic DC4 removed. Multiple images were acquired between the two locations to ensure that no photobleaching was occurring. The same fluorescence emission filter and camera was used for both measurements. The experimental results are presented in Figure 3B together with a fit to the theoretical model with *t*_optics_ as the only free parameter. The value of *t*_optics_ returned was 0.37, which is lower than the value estimated above. A potential explanation for this discrepancy could be due to the manufacturers' reported transmission efficiencies of microscope objectives O2 and O3 being for the case of a uniformly illuminated pupil rather than the case of an isotropically emitting fluorescent object as measured here: isotropic fluorescence emission causes more energy to be concentrated at the edges of the pupil where ray incident angles are higher and anti‐reflection coatings are likely to exhibit reduced performance. The overall relative OPM collection efficiency for an OPM angle of 35° was measured to be 21 ± 1%.

### 3.2 Study of spontaneous Ca^2+^ wave origins from time‐lapse 3‐D OPM imaging

16 cells from 4 AMC animals and 18 cells from 5 HF animals were studied in 3‐D to assess sites of spontaneous Ca^2+^ wave origin. One cell exhibiting almost continuous generation of Ca^2+^ waves was excluded from the study. Table 1 shows the number of spontaneous Ca^2+^ waves recorded. For cells in which at least one wave occurred, the median number of waves per cell was 3.5.

**Table 1:** Summary of the number of cells used for time‐lapse 3‐D imaging of spontaneous calcium waves

	No. of animals	No. of cells	No. of cells with waves	Total no. of waves	No. of waves with origin not overlapping sarcolemma
AMC	4	16	1	1	1
HF	5	18	5	19	14
Total	9	34	6	20	15

**Figure 4:**
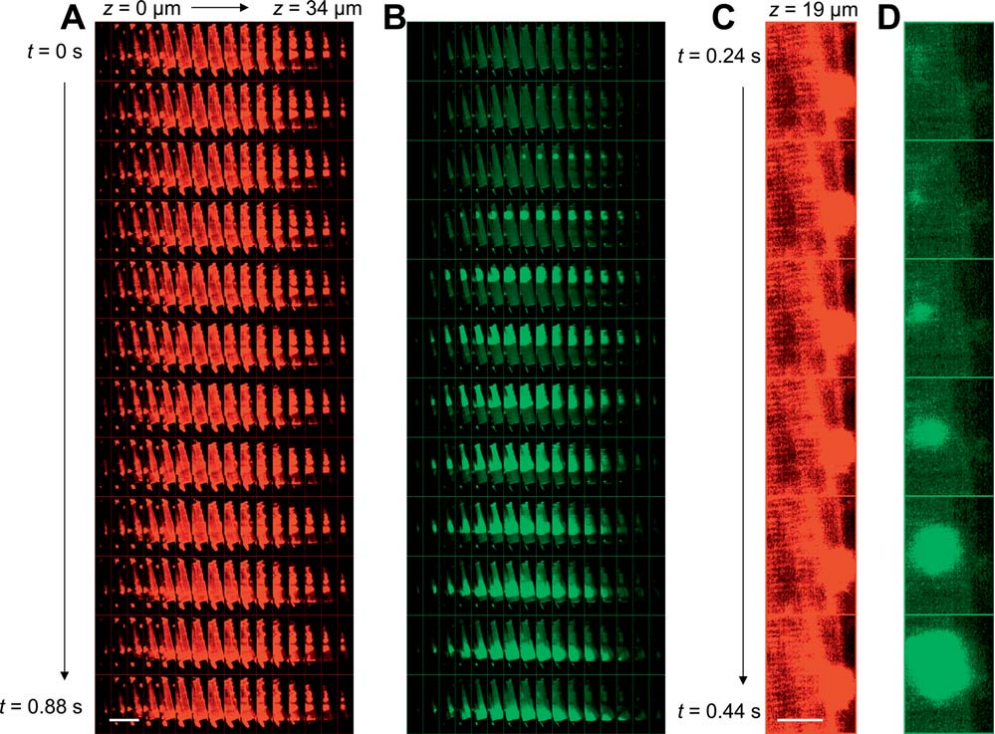
Montages showing time‐lapse 3‐D OPM data of a single spontaneous calcium wave. (**A**) Fluo‐4 and (**B**) CMO montages showing images spaced 2.2 µm apart axially (left to right across montage) and every other acquired volume in time (top to bottom), i.e. rows are 80 ms apart. Scale bar 80 µm. (**C**–**D**) Close‐up of the *z*‐plane containing the spontaneous calcium wave origin. Here, every acquired time‐point is shown, i.e. images are 40 ms apart. Scale bar 13 µm.

Figure 4 and supporting movie S5 show montages of a Ca^2+^ wave recorded by time‐lapse 3‐D OPM imaging. Supporting Figure S6 shows conventional Δ*F*/*F*_0_ traces for two regions of interest within the cell. The spatial origin of the wave can be seen clearly and was located manually in 3‐D. We employed a Fourier analysis approach to measure the periodic modulation of CMO fluorescence intensity due to the *t*‐tubule structure (see Methods) for every voxel within the cell, which we refer to as the *t*‐tubule organization. What this assesses, similar to other measures of *t*‐tubule organization defined in previous Ref. 8, is conformance with a striated staining pattern with tubules running in a transverse direction. As we had determined the position of the origin of the wave in 3‐D, we were therefore able to then lookup the *t*‐tubule organization at that point.

**Figure 5:**
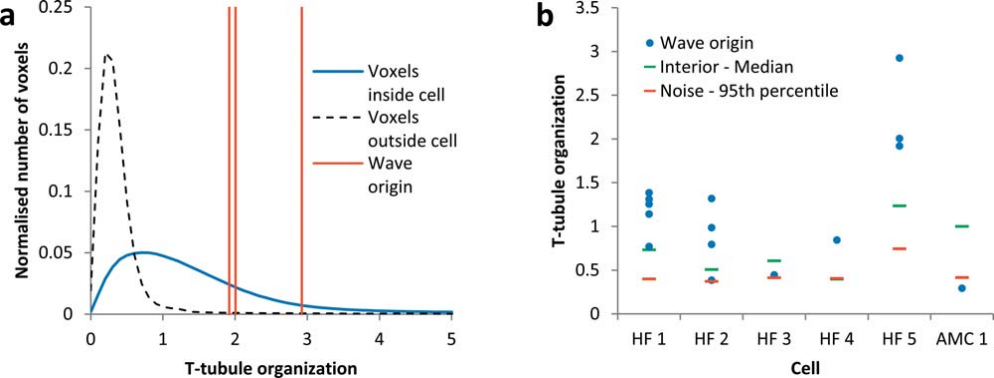
*T*‐tubule organization in relation to wave origin. (**a**) Blue curve shows a histogram of calculated *t*‐tubule organization for all voxels in the interior of cell (same as shown in Figure 2). Black dashed curve shows a histogram of calculated organization for all voxels outside the cell. Red lines indicate the *t*‐tubule organization values obtained at the origin of each spontaneous Ca^2+^ wave observed for this cell. (**b**) *T*‐tubule organization values for each spontaneous Ca^2+^ wave origin for each cell (blue dots). For comparison, the median *t*‐tubule organization from all voxels are also shown for that cell (green bar) and the 95^th^ percentile of the noise for that cell (red bar).

Figure 5a shows an example histogram of the *t*‐tubule organization values for all voxels within one cell (same cell as shown in Figure 4), together with the *t*‐tubule organization values obtained at each of three spontaneous Ca^2+^ wave origins observed within the interior of that cell. For comparison, a histogram of the organization values detected for regions outside the cell, i.e. those organization values generated by noise alone, is also shown (black curve). The black curve provides a lower limit for the ability of the system to detect low *t*‐tubule organization.

Figure 5b shows the *t*‐tubule organization values measured at the origin of each wave for each cell and provides the median *t*‐tubule organization and the 95^th^ percentile of the organization due to noise alone for each cell as comparison. In HF 12 out of 14 wave origins occur at locations with *t*‐tubule organizations greater than the median *t*‐tubule organization for that cell.

If a voxel within a cell is chosen at random, there is a 50% probability of it having a higher *t*‐tubule organization than the median *t*‐tubule organization for that cell. Therefore, using the binomial distribution, the probability of observing by chance 12 or more wave origins with *t*‐tubule organizations greater than the median *t*‐tubule organization for that cell is *p* = 0.0065, indicating that our observation is statistically significant.

### 3.3 Spark data from time‐lapse 2‐D OPM imaging

**Figure 6:**
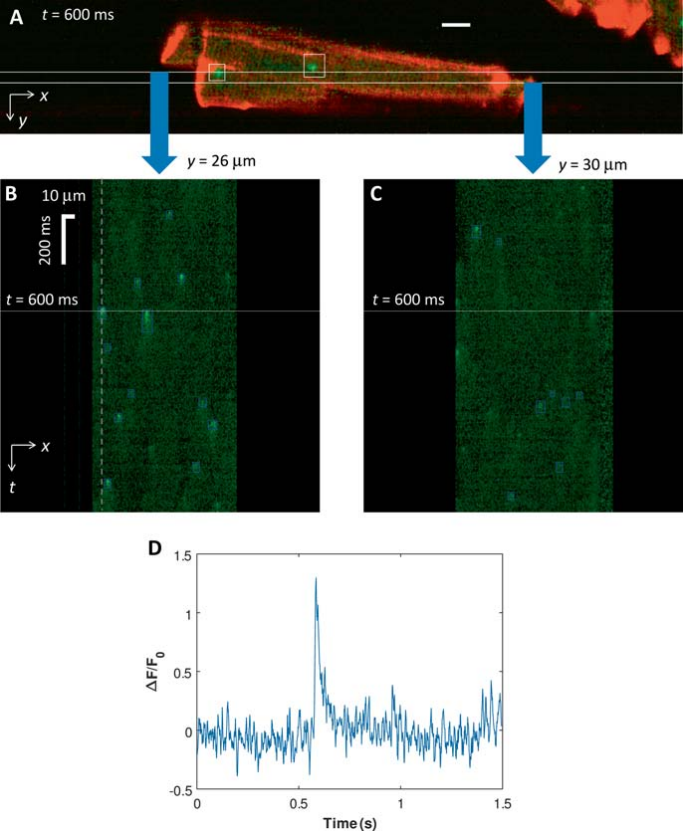
Time‐lapse 2‐D spark imaging with OPM. (**A**) OPM image of a single isolated MI cardiac myocyte acquired at time *t* = 600 ms from the start of image acquisition. Green and red show the fluorescence intensity of Fluo4 and CMO respectively. Scale bar 10 µm. *x*–*t* slices acquired simultaneously through the Fluo4 dataset are shown along the lines at (**B**) *y* = 26 µm and (**C**) *y* = 30 µm. White horizontal lines on line‐scan show 600 ms timepoint corresponding to 2D image. Automatically detected sparks are shown bounded in blue. (**D**) Shows the conventional (*F*(*t*) – *F*_0_)/*F*_0_ = Δ*F*/*F*_0_ trace for the pixel corresponding to the vertical dashed grey line in Figure 6(c). Here *F*_0_ the fluorescence intensity measured at *t* = 0 for this pixel.

Figure 6 illustrates how OPM can be used to image calcium sparks in 2‐D at 667 frames per second. Figures 6B and C show the extent of information available from this form of imaging compared to standard confocal linescan data – an *x*–*t* linescan dataset is available at each *y* co‐ordinate of the cell. Figure 6D shows the conventional Δ*F*/*F*_0_ trace for the pixel corresponding to the vertical dashed grey line in Figure 6C.

In total, 1271 sparks were assessed from 5 myocytes from 3 AMC rats and 3239 from 7 myocytes from 3 HF rats. Spark frequency and morphology was assessed within the different regions of each cell which we define as tubulated (*T_n_* > 0.015) and detubulated (*T_n_* ≤ 0.015). We divided the tubulated region into epitubular (within 0.25 µm of a *t*‐tubule) and paratubular (between *t*‐tubules – further than 0.25 µm).

**Figure 7:**
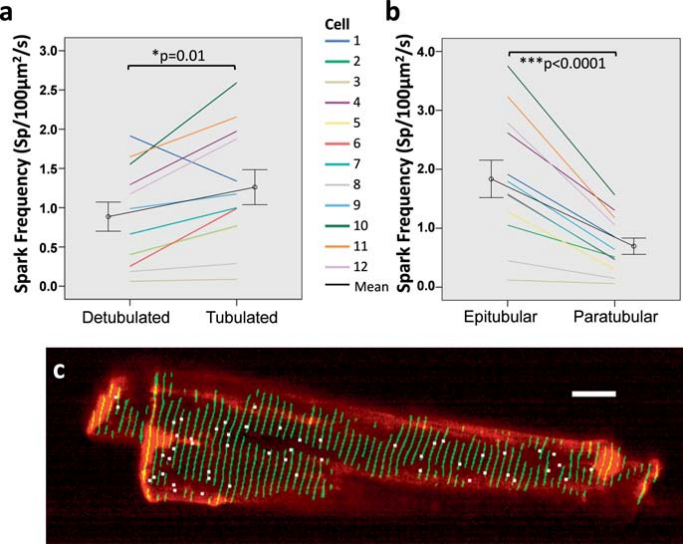
Spark frequency varies according to cell region. (**a**) There is a significant increase in spark frequency in tubulated regions compared to detubulated regions. (**b**) Epitubular regions have significantly higher spark frequency than paratubular regions. (**c**) Map of *t*‐tubule locations (green) and their correspondence to spark location (white dots) during a 1.5 s period in a single HF cell. Scale bar 10 µm. Sparks are localized on top of, or very close to *t*‐tubules with few sparks in paratubular or detubulated regions. Error bars show the standard error.

There were 2 analyses with respect to spark frequency: first, spark frequency was compared between detubulated and tubulated regions; and second, frequency was compared within the tubulated region between epitubular and paratubular sub‐regions. Spark frequency was significantly greater in tubulated compared with detubulated regions (*p* = 0.01, Figure 7a). Within the tubulated region there was a significantly higher spark frequency in epitubular regions compared with paratubular regions (*p* < 0.0001, Figure 7b). This preferential occurrence of sparks at *t*‐tubules within tubulated regions is illustrated by a map of spark occurrence within a single myocyte in Figure 7c.

Spark morphology was then investigated with respect to subcellular region. LogArea was significantly greater in sparks in tubulated regions compared with those in detubulated regions (*p* = 0.007). Within tubulated regions, paratubular sparks had significantly greater area than epitubular sparks (*p* < 0.001). Log_FDHM_ was no different in tubulated regions compared with detubulated regions (*p* = 0.926). However, within tubulated regions, paratubular sparks had significantly greater duration than epitubular sparks (*p* < 0.001).

Using the hierarchical models we assessed if the regional differences depended on whether the cell was from an AMC or HF rat by assessing “interaction” between region and HF. For none of the above measures did this interaction term suggest a difference in relationship between subcellular location and spark frequency/morphology depending on whether the cell was from a HF or AMC rat.

In the limited number of cells studied, the results suggest that in both AMC and HF cells, sparks are more frequent within tubulated regions and in particular in epitubular regions rather than paratubular. In addition spark area is larger in tubulated regions (particularly in paratubular regions) and spark duration is also greater in paratubular regions. Overall these results demonstrate that OPM can be used to measure spark parameters within isolated cardiomyocytes and, importantly, to correlate these parameters with spark spatial location with respect to the *t*‐tubule structure.

## 4 Discussion

### 4.1 Imaging methodology

We have demonstrated that OPM allows imaging of single isolated cardiac myocytes with high temporal resolution in both 2‐D and 3‐D. We have previously demonstrated the potential of OPM to image sparks in isolated cardiomyocytes 17, but significant advances to the system including multiple excitation wavelengths, high resolution and high speed sCMOS cameras and addition of a superfusion system have enabled us to use the system to study the 3‐D origin of spontaneous calcium waves in the context of the *t*‐tubule network for first time.

Although CMO has a track record of *t*‐tubule staining comparable to that of DI‐8‐ANEPPS 28, we found that its combination with Fluo‐4 Ca^2+^ imaging is particularly useful since the overlap of the excitation spectrum of CMO with Fluo‐4 is less than that of DI‐8‐ANNEPS with Fluo‐4. This allows the relative signal levels in the Fluo‐4 and CMO channels to be controlled independently of staining level by varying the relative powers of the excitation beams for the two probes. In addition to the combination of dyes, we have also used a method based on 2‐D Fourier‐domain filtering for assessment of *t*‐tubule organization through the calculation of our *t*‐tubule organization parameter.

### 4.2 OPM of calcium waves in HF

We used a rat HF model that we have previously shown to exhibit enhanced spontaneous SR Ca^2+^ release 29 as well as deranged *t*‐tubule structure 7. We wished to explore if the altered *t*‐tubule organization of these cardiomyocytes influenced where waves originate by locating their origin in 3‐D using CMO to stain the *t*‐tubule network. To our knowledge this is the first example of 3‐D localization of wave origin within a cardiomyocyte. The simultaneous time‐lapse volumetric imaging of both *t*‐tubules and Ca^2+^ allowed us to show in the small number of cells studied (*n* = 5) that in HF waves occur more frequently in regions of organized *t*‐tubule structure than in regions with disorganized *t*‐tubules.

It is conceivable that frequent sparks in the epitubular zones might lead to a particularly high Ca^2+^ in the dyadic space that could result in Ca^2+^ induced Ca^2+^ release (CICR) in adjacent RyR2 clusters and thus initiate the fire‐diffuse‐fire process thought to be responsible for wave propagation 30. The longer, larger sparks in the paratubular zones of well‐organized regions of *t*‐tubules might also produce large rises in dyadic Ca^2+^ and could influence a larger number of surrounding RyR2 clusters to cause internal CICR. Further data is required to confirm or reject this finding and, if confirmed, to determine the mechanisms involved.

### 4.3 OPM of calcium sparks

Using the same microscope in 2‐D mode allows greater temporal resolution and allows us to accurately characterise faster, smaller Ca^2+^ release events (sparks), whilst still considering their position relative to *t*‐tubules.

Previous studies have assessed spontaneous Ca^2+^ spark characteristics in relation to *t*‐tubule structure. Song et al. provided the first exploration of the possibility that subcellular heterogeneity of *t*‐tubules might result in differences in spontaneous SR Ca^2+^ release in their publications identifying “orphaned RyRs” which were not in the vicinity of *t*‐tubules and therefore local dihydropyridine receptors (DHPRs) in HF models 10, 31. They hypothesized that regions of SR containing orphaned RyRs would become Ca^2+^ overloaded and more prone to spontaneous Ca^2+^ sparks. Louch et al. provided experimental data by simultaneously imaging *t*‐tubules and spontaneous Ca^2+^ sparks in control and post‐MI mice using confocal line‐scanning 32. They found that the vast majority (>90%) of sparks, occurred at sites of *t*‐tubules (i.e. equivalent to our epitubular region). They also found that repeating sparks (i.e. those occurring at the same location as an earlier spark) had similar morphologies as the earlier spark, compared with pairs of sparks from different randomly selected locations, suggesting a structural basis for differences in spark morphology. Furthermore in the post‐MI mice there was a population of sparks with increased FDHM (slow sparks). Biesmans et al. also assessed how detubulation might affect spontaneous sparks, using a pig model of ischaemic cardiomyopathy 33. Uncoupled regions (remote from *t*‐tubules) exhibited reduced spark frequency but prolonged spark duration.

Our data also show a significant increase in spark frequency (by a factor of 1.4 – Figure 7a) in tubulated regions compared with detubulated regions. Within regions classified as tubulated, sparks were more frequent in epitubular regions compared with paratubular regions (Figure 7b). There were also significant differences in spark morphology for different regions. Spark area was greater in tubulated compared with detubulated regions. Within regions classified as tubulated, paratubular sparks had a greater area and duration compared with the epitubular sparks. These results could potentially explain why, within an individual cardiomyocyte, Ca^2+^ waves originate from regions in which *t*‐tubules are most prominent, since such regions produce the most frequent, largest and longest Ca^2+^ sparks. With respect to spark morphology data, our findings cannot be compared directly with previous work because sparks were assessed in 2‐D versus 1‐D in previous work and because OPM provides a poorer spatial resolution than confocal microscopy. Nonetheless, all of the results presented in this paper are direct comparisons of spark dimensions between regions within cells and are therefore valid comparisons irrespective of the spatial resolution provided by OPM. In addition, our classification of spark location was more detailed than in previous work including a paratubular category as well as epitubular and detubulated regions.

## 5 Conclusions

We have presented a measurement of the collection efficiency of OPM relative to direct detection of the fluorescence signal in a conventional microscope and shown it to be 21% for an OPM angle of 35° in our setup. In exchange for this lower detection efficiency, OPM has the advantages associated with all light‐sheet microscopy techniques that only the focal plane being imaged is illuminated, i.e. there is no out‐of‐plane photobleaching or phototoxicity and that no image processing or moving parts are required to obtain an optically sectioned image enabling optically sectioned images to be acquired directly at the frame rate of the imaging camera employed.

Through the novel use of OPM we have been able for the first time to localize the origin of Ca^2+^ waves in 3‐D. We have dual‐stained the cells so that the origin is contextualized within the *t*‐tubule structure of the myocyte. We have found in an initial study of a small number of HF cells (*n* = 5) that the greater frequency of waves in HF myocytes originates not from the abnormal detubulated regions but from regions of preserved *t*‐tubule organization. Sparks were also found to be more frequent in regions of high *t*‐tubule organization, particularly in the epitubular zone. The morphology of sparks also varies according to region with larger and more prolonged sparks in paratubular zones. Further studies are required to confirm these results in a larger population of cells.

## Supporting Information

Additional supporting information may be found in the online version of this article at the publisher's website. Original data used in this paper is available under an open source licence through the Open Microscopy Environment: https://cisbic.bioinformatics.ic.ac.uk/omero/webclient/?show=project‐4052.

## Supporting information

Author Biographies

Supporting Information

Calcium Wave OPM Movie
