# Supplementary material for: High speed sCMOS‐based oblique plane microscopy applied to the study of calcium dynamics in cardiac myocytes
Source: J Biophotonics. 2015 Oct 21;9(3):311–23. doi: 10.1002/jbio.201500193 (PMC4874460; doi:10.1002/jbio.201500193)
Supplement: Supplementary file 1 — Author Biographies [file JBIO-9-311-s001.pdf]

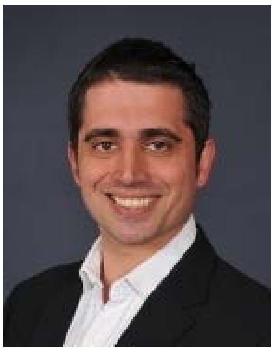

Markus Sikkel graduated from Imperial College School of Medicine in 2004. He attained 1st class honours in an intercalated BSc in pharmacology and toxicology. He has recently completed a PhD on fundamental cellular mechanisms of arrhythmia and is pursuing a career as an academic electrophysiologist.

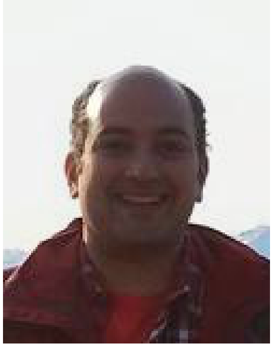

Sunil Kumar completed a BSc degree in Physics in 2002, an MSc in Optics and Photonics in 2004, an MSci in Protein and Membrane Chemical Biology in 2005 and a PhD in 2010 at Imperial College London. Since then, he has worked on fluorescence lifetime imaging in a variety of contexts, primarily high-content screening and Optical Projection Tomography, and also on Oblique Plane Microscopy for high-speed 3D imaging.

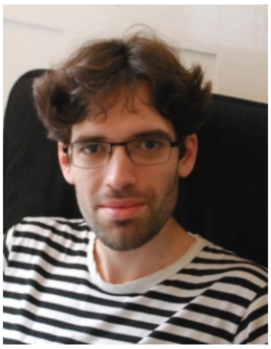

Vincent Maioli studied at the ENS Cachan in France where he completed a BSc in Maths and an MSc in Image processing. After completing an MRes in Photonics at Imperial College London, he is currently in the third year of his PhD there. He is working on Oblique Plane Microscopy for 3D fluorescence imaging.

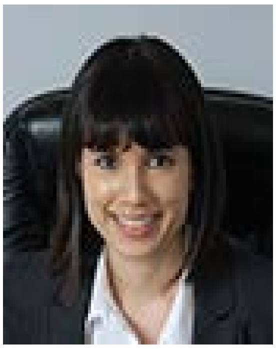

Christina Rowlands received a first class BSc from Royal Holloway in 2010 and was awarded a PhD from Imperial College, London in 2014. Her doctoral research involved the investigation of excitation-contraction coupling in a mouse model of hypertrophic cardiomyopathy prone to sudden cardiac death. She is now a research associate for a healthcare consulting company.

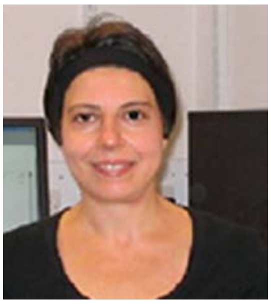

Fabiana Gordon is a Senior Statistical Consultant at the Statistical Advisory Service of Imperial College London.

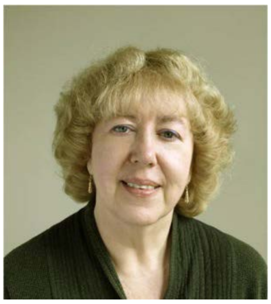

Sian Harding is Professor of Cardiac Pharmacology at Imperial College London and Director of the Imperial British Heart Foundation Cardiovascular Regenerative Medicine Centre. Her work has centered on the myocardium in heart failure, especially beta-adrenergic mechanisms. She is PI on the first UK Gene therapy Trial in LVAD patients, aimed at improving cardiac contractility. She is now studying the pluripotent stem cell-derived cardiomyocytes, both for disease modelling and cardiac repair. Professor Harding is former President of the European Section of the International Society for Heart Research and member of the Nuffield Council on Bioethics. She has been elected Fellow of both the American Heart Association and the European Society of Cardiology, and is on the Board of the British Society of Gene and Cell Therapy.

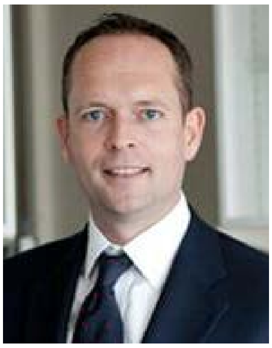

Alexander Lyon is a Senior Lecturer in Cardiology at Imperial College, London and a Consultant Cardiologist at the Royal Brompton Hospital. His clinical and research interests are in the field of heart failure, chemotherapy cardiomyopathy and Takotsubo cardiomyopathy, and he is the theme leader for heart failure research in the NIHR-funded Biomedical Research Unit at the Royal Brompton Hospital. He is currently president of the British Cardio-Oncology Society.

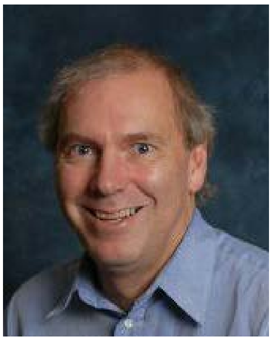

Ken MacLeod is currently Reader in Cardiac Physiology at Imperial College. He obtained his degrees at Aberdeen and Edinburgh Universities and did post-doctoral work at University of California. His laboratory examines the processes that control cardiac cell contraction in health and disease. Investigation of these processes are fundamental to our understanding of the workings of the heart, will allow a more logical approach to therapy and, in the longer term, may provide impetus for the generation of novel treatments. The processes are studied at a variety of biological levels, from the single cell to the whole heart using a variety of physiological and biophysical techniques.

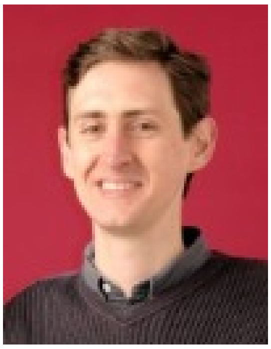

Chris Dunsby received an MSci. degree from Bristol University in 2000. In 2003 he received a Ph.D. from Imperial College, UK, in "Wide-field Coherence-gated Imaging Techniques Including Photorefractive Holography". He is now a reader with a joint post between Photonics, Department of Physics and the Centre for Histopathology, Department of Medicine at Imperial. His research interests are centred on the application of photonics and ultrafast laser technology to biomedical imaging and include multiphoton microscopy, multiparameter fluorescence imaging and fluorescence lifetime imaging.
