# Supplementary material for: High speed sCMOS‐based oblique plane microscopy applied to the study of calcium dynamics in cardiac myocytes
Source: J Biophotonics. 2015 Oct 21;9(3):311–23. doi: 10.1002/jbio.201500193 (PMC4874460; doi:10.1002/jbio.201500193)
Supplement: Supplementary file 2 — Supporting Information [file JBIO-9-311-s002.pdf]

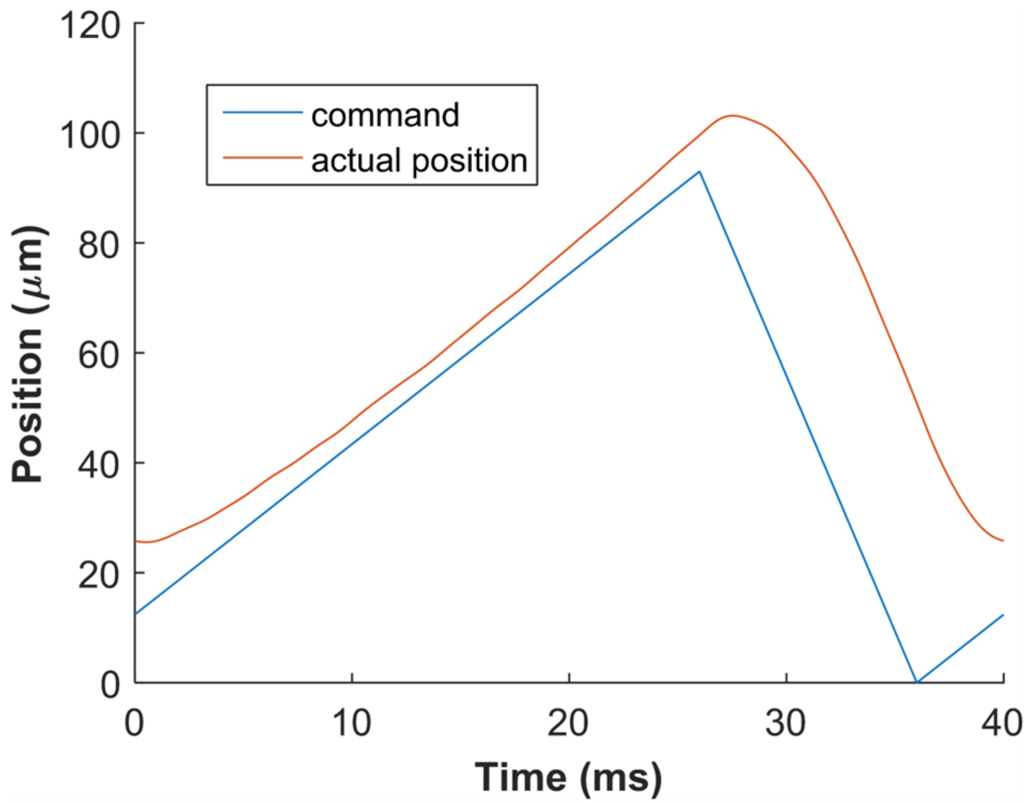

S1 Figure. Example plot showing commanded piezo actuator position and actual actuator position recorded by the capacitive position sensor during time-lapse 3-D image acquisition.

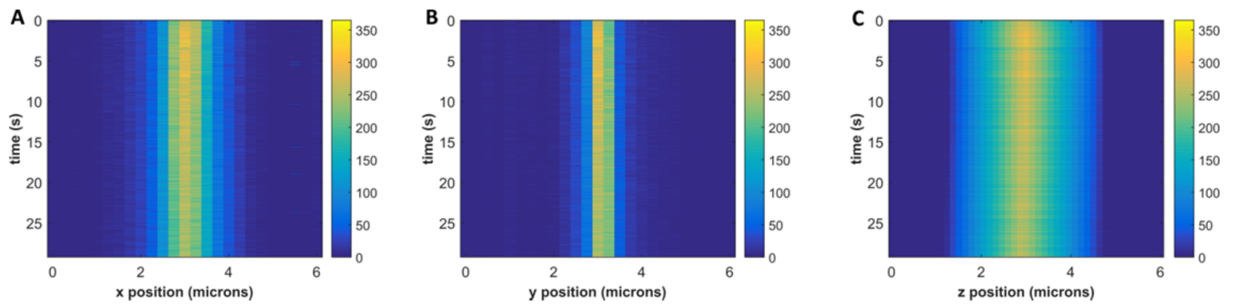

S2 Figure. OPM system stability measured from a time-lapse 3-D volume (dimensions *x*,*y*,*z*,*t*) of a single 200 nm fluorescent bead. The acquisition was performed at 25 volumes per second over an axial scan range of O2 of 77 µm with the same acquisition parameters as the time-lapse 3-D imaging of the cardiac myocytes. From the resulting 4-D data set we took 2-D plots through the centre of the bead to illustrate the stability of the system during image acquisition. a) *x*,*t*, b) *y*,*t*, c) *z*,*t* plots through the centre of the bead. Here *x* and *y* are orthogonal and in the plane of the illumination sheet with *x* parallel to the light sheet illumination direction. *z* is perpendicular to the illumination sheet.

S3 - Details of spark detection algorithm

The 2-D time-lapse OPM data was analysed in sections of 1000 frames that were chosen to exclude the presence of Ca^{2+} transients and waves. The algorithm was as follows:

1) Pre-processing. The camera digital offset and background light were measured from a dark corner of the image and subtracted. The *x-y-t* data was then smoothed spatially and temporally using a 3×3×5 kernel.

2) Identification of preliminary sparks. The initial estimate of the mean, μ_1, and standard deviation, σ_1, of each *x-y* point in the image stack were calculated along the *t* axis. Preliminary spark *x-y-t* regions were identified according to two criteria. The first criterion was that all pixels within a region are more than $3.2\sigma_1$ above μ_1. The second criterion was that the region must contain at least one pixel that is more than $6\sigma_1$ above μ_1.

3) Calculation of the fractional change in fluorescence intensity $\Delta F/F_0$, where $\Delta F= F - F_0$. Preliminary sparks (identified in step 2) were removed from the data. Then the first 30 frames of each 1000 frame section were averaged to find the fluorescence intensity at the start of the section, F_0. The fractional change in fluorescence intensity $\Delta F/F_0$ was then calculated for each pixel of the *x-y-t* dataset.

4) Identification of potential sparks. The mean, μ_2, and standard deviation, σ_2, of the fractional change in fluorescence intensity – excluding preliminary sparks – was calculated along the *t* axis for each x-y point in the image stack. Potential sparks were defined as *x-y-t* regions meeting the first and second criteria used in step 2.

5) Identification of actual sparks. The area of each potential spark was calculated using the maximum *x-y* extent of the spark as determined using the first criterion. Similarly, the duration of the spark was calculated as the temporal extent of the spark region as determined using the first criterion. Sparks with an area below 3 μm^2 and a full duration (at the level defined by the first criterion) below 18 ms were excluded.

6) Actual spark parameters. The following parameters were calculated for actual sparks: spark amplitude in terms of maximum fractional change in fluorescence intensity $\Delta F/F_0$; spark area measured at the border defined by the first criterion; spark full duration at half maximum (FDHM); spark centre of mass in *x* and *y*; normalised t-tubule modulation at spark centre of mass; and distance of spark centre of mass to a region defined as a t-tubule.

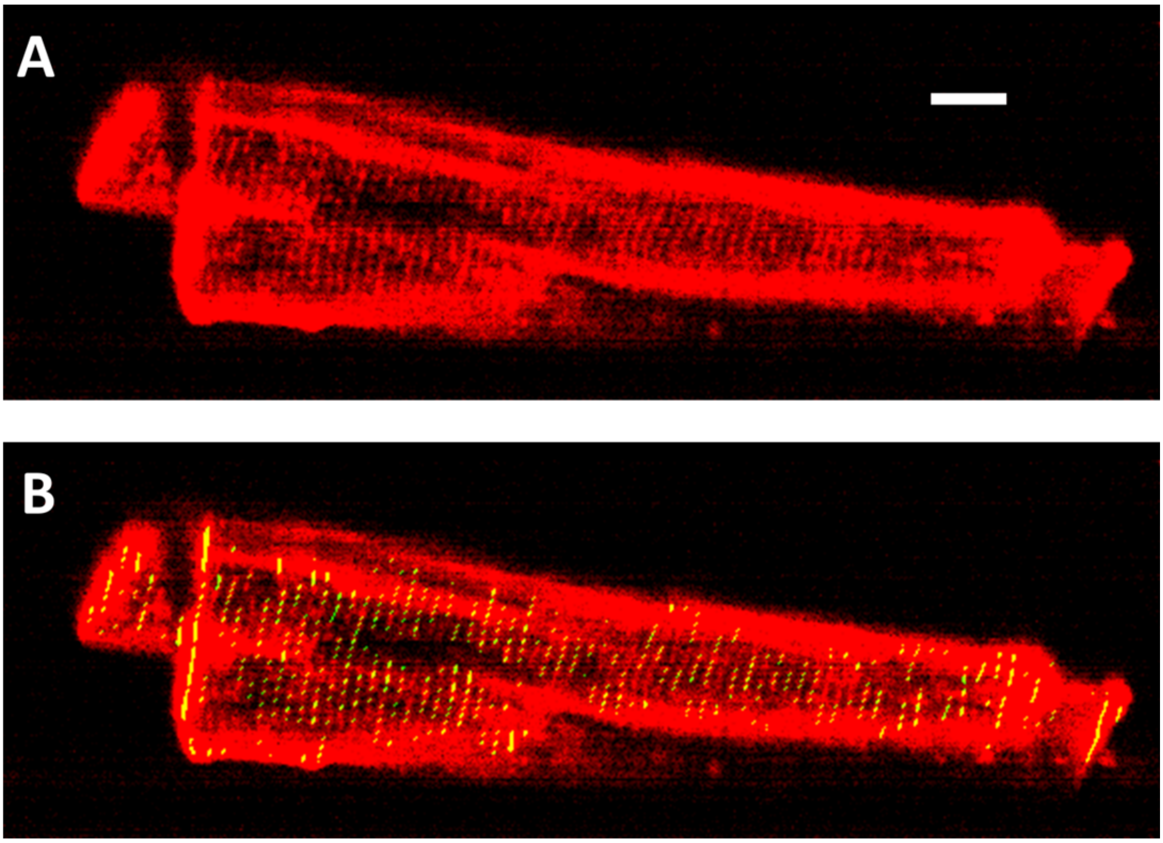

S4 Figure. Illustration of the t-tubule localisation algorithm showing the alignment of the t-tubules in the original CMO image relative to the t-tubules detected by the t-tubule detection algorithm. a) CMO image (red) and b) same image overlayed with the output of the t-tubule detection algorithm (green) eroded to enable the original tubulation to be seen underneath. The brightness and contrast of both channels have been adjusted to enable them to be compared more easily. Scale bar 10 µm.

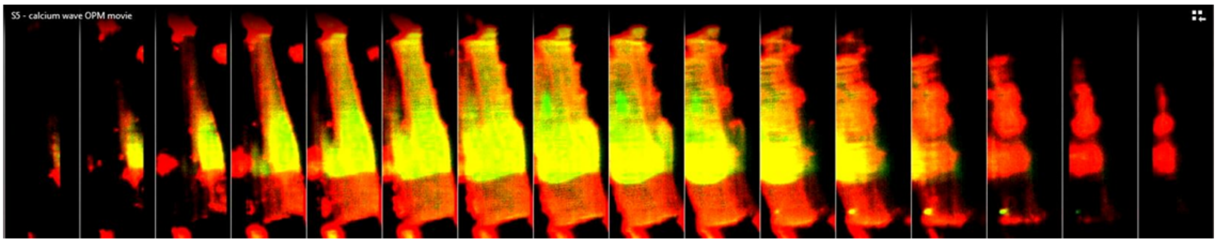

(Screenshot from video)

S5 Movie. Video of time-lapse 3-D OPM data of spontaneous calcium wave origin. Montage shows images spaced 2.2 µm apart axially (left to right across montage) with Fluo-4 shown in green and CMO in red. Volumes were acquired 40 ms apart. Scale bar 80 µm.

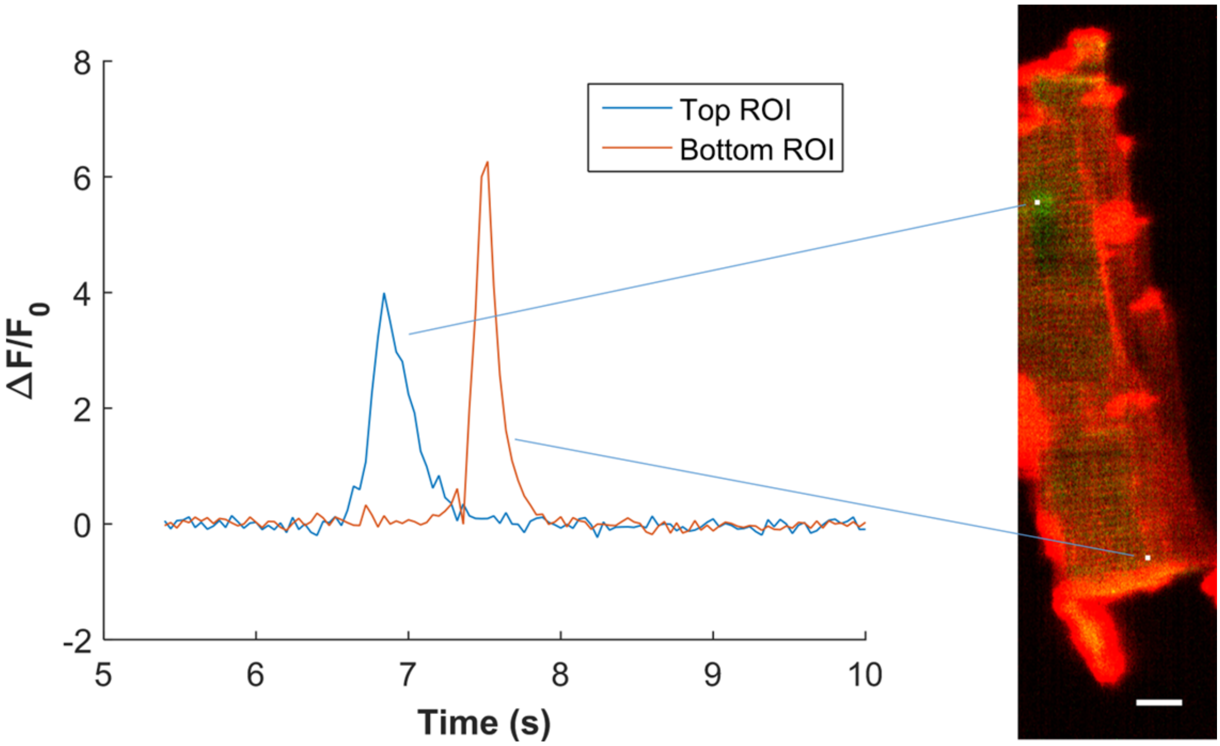

S6 Figure. Line profiles of the fluorescence intensity in the Fluo4 channel as a function of time for two 5×5 pixel regions of the cell indicated in the inset. Data shown is the same as that in figure 4 and from the plane $z = 21$ µm. Values are plotted as $(F(t)\text{-}F_0)/F_0 = \Delta F/F_0$ where F_0 is the fluorescence intensity measured for each region at the start of each trace. We note that the second ROI at the bottom of the image is situated in a region of the cell experiencing motion due to the contraction of the cell, as can be seen in supporting information movie S5. Scale bar 10 µm.
